# Celiac crisis, transient hypertransaminasemia and positive smooth muscle antibodies (SMA): A celiac disease case report

**DOI:** 10.1002/jpr3.12056

**Published:** 2024-02-22

**Authors:** Federica Rodofile, Paola Melli, Andrea Di Siena, Paola Cogo

**Affiliations:** ^1^ Division of Pediatrics, Department of Medicine (DAME) University of Udine Udine Italy

**Keywords:** anti‐actin antibodies, autoimmune hepatitis, liver injury

## Abstract

Celiac crisis (CC) is a rare complication of celiac disease (CD), usually observed in younger children with unrecognized CD or poor compliance with a gluten‐free diet (GFD). We present a case of celiac crisis in a 3‐year‐old girl who was recently diagnosed with celiac disease. She was referred to our clinic with anasarca, tetany, and severe malnutrition, with hypokalemia, hypocalcemia, hypomagnesemia, and hypoalbuminemia. During hospitalization, she presented hypertransaminasemia with positive anti‐actin smooth muscle antibodies (SMA). Abdominal ultrasound and liver biopsy were normal, excluding autoimmune hepatitis. Liver involvement is a common CD extraintestinal manifestation and cryptogenic form is the most common. SMA positivity could be associated with a systemic immune cross reaction. Our patient normalized liver values after 2 months of GFD.

## Abstract (highlights)

**What is Known:**

- Celiac disease (CD) is a commonly diagnosed pediatric disorder that may present with celiac crisis (CC), a relatively uncommon occurrence.
- The onset of CD may also be associated with liver injuries and, occasionally, autoimmune hepatitis (AIH). AIH is suspected through the detection of specific antibodies, smooth muscle antibodies (SMA), and hypertransaminasemia.

**What is New:**

- In our case, CC demonstrated transient hepatic injury, as indicated by a transient hypertransaminasemia, followed by rapid normalization.
- CD triggers immune system activation, leading to the development of specific antibodies typically associated with liver disease. In certain instances, as in our patient, positive F‐actin SMA antibodies in CD may result from a cross‐reaction mechanism. Alternatively, it may be linked to villous intestinal atrophy.

**Translational impact:**

- Given the prevalence of CD, understanding its potential rare complications is crucial.
- In cases of hypertransaminasemia and positive SMA antibodies, particularly of the anti‐actin type, consideration for undiagnosed CD is recommended.

## 1 INTRODUCTION

Celiac disease (CD) is an immune‐mediated systemic disorder elicited by gluten in genetically susceptible individuals and is characterized by a variable combination of clinical manifestations, CD‐specific antibodies, and enteropathy with subsequent nutritional malabsorption.^1^ In Italy, the prevalence of CD in children is 1.65%.^2^ Celiac crisis (CC) is a rare complication of CD, is most frequent in early childhood, and is often triggered by intestinal infectious diseases.^3^ CC could be a complication of unrecognized CD or a consequence of noncompliance with the recommended gluten‐free diet (GFD). Liver involvement is a common manifestation of CD: isolated hypertransaminasemia is frequent, but in some cases, cryptogenic cirrhosis and liver transplant are reported.^4^ We present a case of a 3‐year‐old girl with CC and suspected autoimmune hepatitis in a recent CD diagnosis.

## 2 MATERIALS AND METHODS

We performed a literature search of other similar cases.

## 3 CASE PRESENTATION

**Figure 1:**
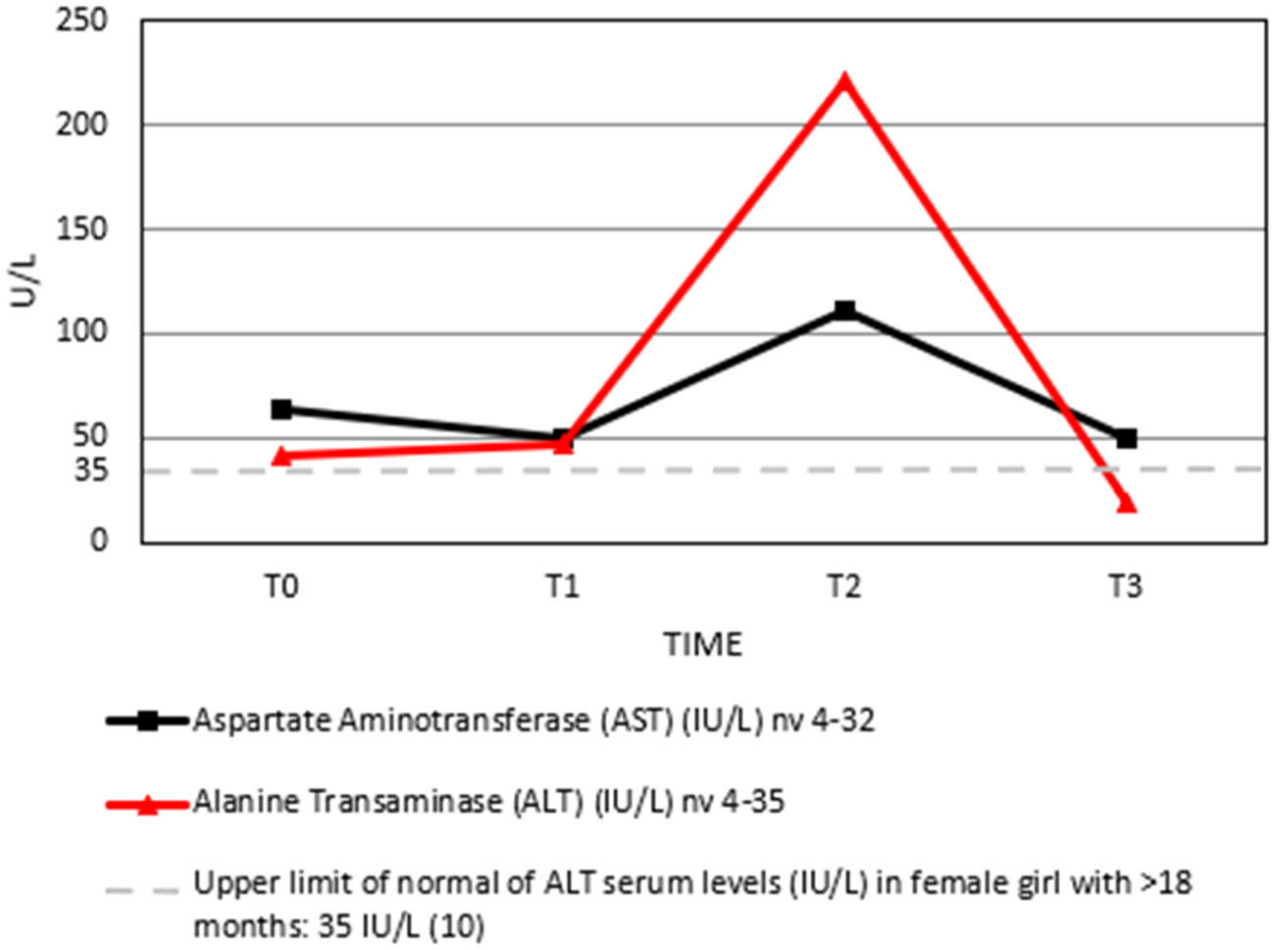
Aspartate aminotransferase (AST) and alanine transaminase (ALT) trend in our patient. Values at admission time (T0); values 3 days after admission (T1); values 2 weeks after the admission (T2); values 2 months after admission (T3).

We present the case of a 3‐year‐old girl admitted to our emergency department because of CC. She had a 1‐month history of diarrhea and asthenia, so a few days before, she performed stool microbiological tests resulted negative and blood tests that resulted positive for IgA anti‐tissue transglutaminase antibodies (anti‐TG2) (8200 UA/mL, cut‐off <7.0 U/mL) and IgA anti‐endomysial (EMA) antibodies at high titers in a second blood sample. She presented high level values at the calprotectin test. Therefore, she was diagnosed with CD according to the recent European Society for Pediatric Gastroenterology, Hepatology and Nutrition (ESPGHAN), with no‐biopsy approach, and she started a GFD.^1^ A few days later, she was referred to our clinic with anasarca, tetany and weight loss. The first blood tests revealed neutropenia (neutrophil count: 150/mmc) and severe malnutrition with typical signs of CC such as hypokalemia, hypocalcemia, hypomagnesemia and hypoalbuminemia (see Table 1). She performed an infusion to correct electrolyte imbalance. Autoimmune thyroiditis, autoimmune neutropenia and immunological deficits were excluded: lymphocyte subpopulations and IgG, IgM and IgA immunoglobulins were normal. A few days after hospitalization, she presented a progressive hypertransaminasemia (Figure 1) with maximum values of aspartate aminotransferase (AST) 111 U/L and alanine transaminase (ALT) 221 U/L, with normal gamma‐glutamyl transferase (GGT) value. She performed an abdominal ultrasound that did not show hepatosplenomegaly or hepatic injuries. Smooth muscle antibodies (SMA) resulted positive (title 1:320, vessels‐glomerular‐tubular (VGT) immunofluorescence pattern, anti‐actin pattern) and, in the suspicion of autoimmune hepatitis (AIH), a liver biopsy was performed. The results of the biopsy allowed us to exclude liver injuries and autoimmune hepatitis. Transaminase values normalized 2 months after starting GFD (Table 1).

**Table 1:** Blood tests showed severe acute malnutrition (electrolytes disorders, folate and vitamin D deficiency, prolonged INR caused by vitamin K deficiency, etc.), liver injuries and transient hypertransaminasemia.

		Unit of measurement	Values at admission time	Values 3 days after admission	Values 2 months after admission	Normal laboratory values
White cells (/mmc)	Total	cells/mmc	4750	4920	5750	5500–15,000
	Neutrophils	cells/mmc	380	150	1680	1300–8500
Hemoglobin		g/dL	8.6	8.9	11.4	11–13.5
Albumin		g/L	25	27	43	38–54
Protein		g/L	42			64–83
Sodium		mmol/L	139	142	141	135–145
Potassium		mmol/L	2.88	3.46	4.4	3.5–5.1
Total calcium		mmol/L	1.2		2.44	2.2–2.7
Calcium ion		mmol/L	0.73	1.27		1.15–1.27
Magnesium		mmol/L	0.21	0.55	0.89	0.7–0.95
Phosphorus		mmol/L	1.04	0.98	1.66	1.05–1.8
Aspartate aminotransferase (AST)		U/L	63	50 (max value 111)	32	4–32
Alanine transaminase (ALT)		U/L	42	47 (max value 221)	17	4–35
Gamma‐glutamyl transferase (GGT)		U/L	6		7	5–55
Total bilirubin		mg/dL	0.06		0.10	0.2–1
Direct bilirubin		mg/dL	0.03		0.01	0.00–0.3
Vitamine D		ng/mL	21			30–100
Vitamine B_12_		pg/mL	420			211–911
Folate		ng/mL	3			3–17
Cupper		umol/L	9.5			10.2–20.5
Zinc		umol/L	8.2			9–15.7
INR				1.21		0.8–1.1

## 4 DISCUSSION

Our patient presented with CC with severe nutritional deficiencies, malnutrition, and hypertransaminasemia related to CD. Our patient also met moderate‐severe criteria for refeeding syndrome (−2.9 z‐score, weight loss, electrolyte imbalance, etc.) according to the American Society for Parenteral and Enteral Nutrition (ASPEN),^5^ which may be associated with CC, as reported in the literature.^6^ Liver involvement is a common extraintestinal manifestation from mild hepatic injury to severe liver disease. Liver can be involved in two main forms: cryptogenic and autoimmune disease. The first type is frequent. In a recent meta‐analysis, Sainsbury et al.^7^ estimated the prevalence of CD in adults with cryptogenic hypertransaminasemia and the prevalence of hypertransaminasemia in patients with a new CD diagnosis. The combined proportion with positive celiac serology and biopsy‐proven CD in unexplained hypertransaminasemia were 6% and 4%, respectively. Approximately one‐third of patients newly diagnosed with CD had hypertransaminasemia.^7^ Iorio et al. investigated 425 children with isolated hypertransaminasemia.^8^ Three of 166 patients (1.8%) with persistent hypertransaminasemia were identified as having CD. This is about two times the risk of CD in the general population. So, a routine laboratory screening for CD in patients with unexplained hypertransaminasemia can be useful in diagnosing CD in the absence of other symptoms. Even in children hypertransaminasemia may represent the only manifestation of CD, in fact about 40% of children had isolated hypertransaminasemia during diagnostic analysis for suspected CD. In our case, biochemical liver tests normalized after 2 months of GFD. The pathogenesis of the hypertransaminasemia and liver damage in CD remains currently unknown. AIH is less frequently diagnosed. Other rare types of liver damage in CD are primary sclerosing cholangitis and fatty liver disease. In our case, we perform a liver biopsy due to positive serology SMA and to exclude AIH. Liver biopsy was normal and transaminase values normalized after 2 months of GFD. SMA antibodies are a heterogenous group of antibodies directed against cytoskeleton components, found in liver diseases, viral infections, autoimmune pathologies and tumor cells.^9^ Infectious diseases were not further investigated considering the recent diagnosis of CD and the absence of symptoms and laboratory tests suggestive of viral infection, such as white blood cells and lymphocytes in range, negative C‐reactive protein and erythrocyte sedimentation rate both 2 weeks before the onset of CC and during the hospitalization. Bottazzo et al. described the distribution of specific SMA variants in liver diseases (V‐G‐T: vessels, glomeruli, tubuli) and the association between SMA T/G antibodies at higher titers (greater than 1:80) and AIH.^10^ Anti‐actin SMA antibodies are most specific for type 1 AIH but they are also described in CD (in particular F‐actin pattern) and appear to be related to the presence of intestinal villus atrophy and to more severe clinical CD outcome, so Clemente et al. suggested that it could be used as a marker of severe intestinal atrophy.^11^ Granito et al. stressed the possibility of cross‐linking between the actin filaments of the cytoskeleton with tTG, as actin is a good substrate of it. Indeed, the cross‐linking could explain a hypothetical mechanism of secondary autoimmune phenomena related to CD.^12^ Our patient did not perform an intestinal biopsy to evaluate villous atrophy but the onset with CC and the presence of actin IgA and SMA VGT at high title raise suspicion of important intestinal injuries.

## 5 CONCLUSION

CC is a rare onset of CD, and it could be triggered by an immune activation caused by a general immune stimulus (infectious diseases, trauma, or untreated CD as in our patient). Our girl presented typical CC symptoms but also hypertransaminasemia and specific liver diseases antibodies. We performed transaminase monitoring and liver biopsy on suspicion of AIH in consideration of the clinic and blood tests. The results showed that AIH was not confirmed, so SMA positivity could be associated with an immune cross reaction. Although more and more detailed techniques, such as specific methods of indirect immunofluorescence, western blot and ELISA tests, are being used to look for the most specific antibodies to every different type of liver disease, it is still not possible to rely on serology to rule out AIH, making it necessary to perform a liver biopsy.

## CONFLICT OF INTEREST STATEMENT

The authors declare no conflicts of interest.

## References

[1] Husby S , Koletzko S , Korponay‐Szabó I , et al. European society paediatric gastroenterology, hepatology and nutrition guidelines for diagnosing coeliac disease 2020. J Pediatr Gastroenterol Nutr. 2020;70(1):141‐156. 10.1097/MPG.0000000000002497 31568151

[2] Lionetti E , Pjetraj D , Gatti S , et al. Prevalence and detection rate of celiac disease in Italy: results of a SIGENP multicenter screening in school‐age children. Dig Liver Dis. 2023;55(5):608‐613. 10.1016/j.dld.2022.12.023 36682923

[3] Radlovic N , Lekovic Z , Radlovic V , et al. Celiac crisis in children in Serbia. Ital J Pediatr. 2016;42:25. 10.1186/s13052-016-0233-z 26931303 PMC4774094

[4] Casswall TH , Papadogiannakis N , Ghazi S , Németh A . Severe liver damage associated with celiac disease: findings in six toddler‐aged girls. Eur J Gastroenterol Hepatol. 2009;21:452‐459. 19182681 10.1097/MEG.0b013e32830e1f12

[5] da Silva JSV , Seres DS , Sabino K , et al. ASPEN consensus recommendations for refeeding syndrome. Nutr Clin Pract. 2020;35(2):178‐195. 10.1002/ncp.10474 32115791

[6] Catassi C . Celiac Crisis/refeeding syndrome combination: new mechanism for an old complication. J Pediatr Gastroenterol Nutr. 2012;54(4):442‐443. 10.1097/MPG.0b013e318242fe3a 22142986

[7] Sainsbury A , Sanders DS , Ford AC . Meta‐analysis: coeliac disease and hypertransaminasaemia. Aliment Pharmacol Ther. 2011;34(1):33‐40. 10.1111/j.1365-2036.2011.04685.x 21545472

[8] Iorio R , Sepe A , Giannattasio A , Cirillo F , Vegnente A . Hypertransaminasemia in childhood as a marker of genetic liver disorders. J Gastroenterol. 2005;40:820‐826. 10.1007/s00535-005-1635-7 16143887

[9] Selmp C , Muratori P , Podda M , Bianchi FB . Smooth muscle antibodies. In: Yehuda Shoenfeld M , Gershwin E , Meroni PL , eds. Autoantibodies. 2nd ed. Elsevier; 2007:487‐491. 10.1016/B978-044452763-9/50065-2

[10] Bottazzo GF , Florin‐Christensen A , Fairfax A , Swana G , Doniach D , Groeschel‐Stewart U . Classification of smooth muscle autoantibodies detected by immunofluorescence. J Clin Pathol. 1976;29:403‐410. 777046 10.1136/jcp.29.5.403 PMC476077

[11] Clemente MG . Immune reaction against the cytoskeleton in coeliac disease. Gut. 2000;47:520‐526. 10986212 10.1136/gut.47.4.520 PMC1728086

[12] Granito A , Muratori P , Cassani F , et al. Anti‐actin IgA antibodies in severe coeliac disease. Clin Exp Immunol. 2004;137(2):386‐392. 10.1111/j.1365-2249.2004.02541.x 15270857 PMC1809109

